# Online Medical Consultation Service–Oriented Recommendations: Systematic Review

**DOI:** 10.2196/46073

**Published:** 2024-07-30

**Authors:** Hongxun Jiang, Ziyue Mi, Wei Xu

**Affiliations:** 1 School of Information, Renmin University of China Beijing China; 2 School of Smart Governance, Renmin University of China Suzhou China

**Keywords:** online health community, online medical consultation, personalized recommendations, 2-sided matching, load balancing

## Abstract

**Background:**

Online health communities have given rise to a new e-service known as online medical consultations (OMCs), enabling remote interactions between physicians and patients. To address challenges, such as patient information overload and uneven distribution of physician visits, online health communities should develop OMC-oriented recommenders.

**Objective:**

We aimed to comprehensively investigate what paradigms lead to the success of OMC-oriented recommendations.

**Methods:**

A literature search was conducted through e-databases, including PubMed, ACM Digital Library, Springer, and ScienceDirect, from January 2011 to December 2023. This review included all papers directly and indirectly related to the topic of health care–related recommendations for online services.

**Results:**

The search identified 611 articles, of which 26 (4.3%) met the inclusion criteria. Despite the growing academic interest in OMC recommendations, there remains a lack of consensus among researchers on the definition of e-service–oriented recommenders. The discussion highlighted 3 key factors influencing recommender success: features, algorithms, and metrics. It advocated for moving beyond traditional e-commerce–oriented recommenders to establish an innovative theoretical framework for e-service–oriented recommenders and addresses critical technical issues regarding 2-sided personalized recommendations.

**Conclusions:**

This review underscores the essence of e-services, particularly in knowledge- and labor-intensive domains such as OMCs, where patients seek interpretable recommendations due to their lack of domain knowledge and physicians must balance their energy levels to avoid overworking. Our study’s findings shed light on the importance of customizing e-service–oriented personalized recommendations to meet the distinct expectations of 2-sided users considering their cognitive abilities, decision-making perspectives, and preferences. To achieve this, a paradigm shift is essential to develop unique attributes and explore distinct content tailored for both parties involved.

## Introduction

### Background

Technology innovations have brought the medical industry into the digital, networked, and intelligent era of the medical internet. Combined with the impact of the pandemic, telemedicine increasingly prevails. A growing number of patients have started to seek contactless counseling from physicians through online health communities (OHCs). Meanwhile, physicians are providing the public with health care posts, free medical consultations, and even paid customized service [1-3]. HaoDF, one of the leading OHC platforms in China, has gathered >890,000 physicians from 10,000 hospitals across the country up to March 2023. It offers patients the service of telehealth or web-based live chat (ie, online medical consultation [OMC]). Telehealth offers greater convenience to patients than the in-person visits previously available. However, it worsens the problem of information overload, as there are too many candidates for users to choose from, which exacerbates the level of hesitation [4]. Patients face challenges in selecting suitable physicians due to limited medical knowledge and cognitive abilities. An OMC-oriented recommendation system is crucial to provide patients with professional, accurate, and responsible referrals, ensuring that they connect with qualified and suitable physicians.

**Figure 1:**
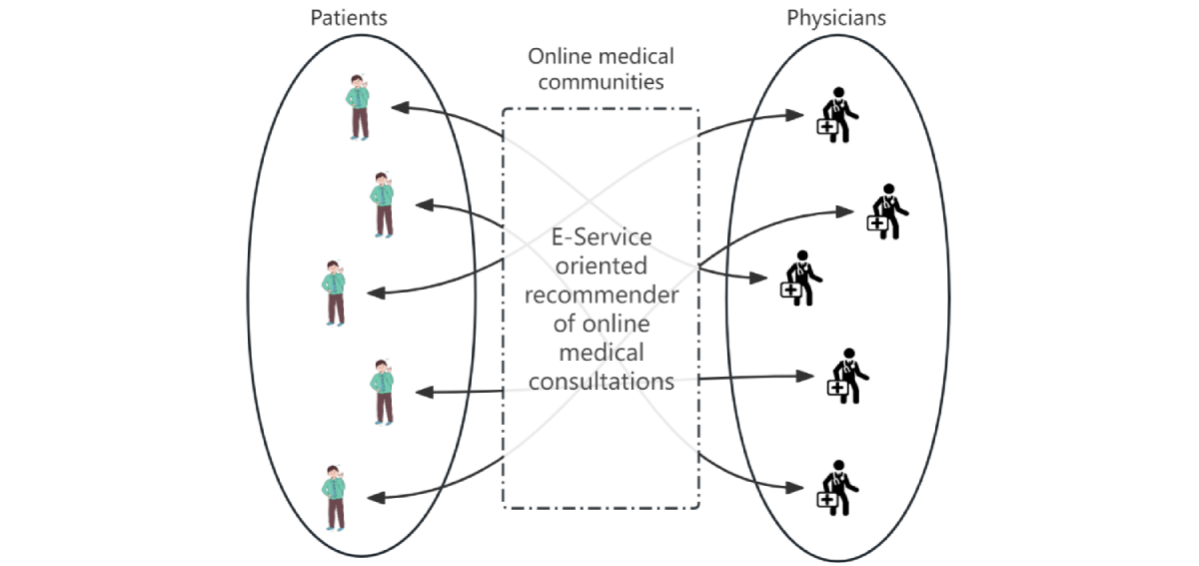
Online health communities function as 2-sided markets involving both patients and physicians. In such markets, the user base on one side of the platform affects the user base on the other side, leading to cross-network externalities that influence the overall transaction volume across both sides. Recommendation systems must consider both patients’ and physicians’ needs and preferences.

Most existing studies of physician recommendations are in the wrong direction regardless of their diverse methodologies, such as collaborative filtering (CF), demographic statistics, or association rules. Previous research has overlooked the fact that OHCs serve both patients and physicians (ie, a 2-sided market scenario). Figure 1 illustrates an OHC jointly formed by patients and physicians. When a market is 2-sided, there are cross-network externalities, which means that the number of users on one side will affect the number of users on the other side and the overall transaction volume on both sides [5]. An OMC recommendation is a service that an OHC offers to both sides of users. A type of online service such as OMC (ie, an e-service) is an emerging field of internet business under the knowledge economy. As opposed to e-commerce, an e-service is composed of consultees and consultants rather than users and commodities. The offered item is an intangible service rather than a tangible one, but it has to meet the different needs, expectations, and preferences of 2-sided users. Furthermore, medical consultations are knowledge- and labor-intensive services that demand high levels of professionalism and energy investment [6]. The energy limits of physicians vary, and each physician can receive consultations only to a certain extent. In addition, patients lack the professional knowledge to distinguish the candidates, so they need recommendations that can be interpreted. Thus, it is impossible to transplant an e-commerce recommendation model to solve OMC recommendation cases. Research on recommendation systems suffers from a “blind side” that is the lack of research focusing on service-oriented applications, requiring academicians to develop new attributes and research new content. OMC services demonstrate the typical characteristics of online knowledge services, which represent the emerging trend of the “Internet+” economy. In the context of the knowledge economy, research on service recommendations is particularly pertinent, and now is an excellent time to start. As far as we know, no comprehensive research has been conducted in academic circles on service recommendations. Personalized service recommendation is a new topic yet to be clearly defined and fully explored.

### Objectives

Despite several reviews on health care recommender systems focusing on patient interests [7-9], there remains a gap in service-oriented recommendations. Our systematic review aimed to fill this void by concentrating solely on 2-sided recommendations. By providing the latest review of this domain, we aimed to gather comprehensive evidence for evaluating current studies, identifying successful paradigms and approaches in service-oriented recommendations, and informing public health interventions and policy making. This will leverage 2-sided recommendation technologies to enhance the well-being of both patients and physicians in the emerging OMC service industry.

## Methods

This review was conducted according to the PRISMA (Preferred Reporting Items for Systematic Reviews and Meta-Analyses) guidelines (Multimedia Appendix 1 [10]) [11].

### Search Strategy

As the OMC service–oriented recommendation system spans multiple disciplines such as health care, business information systems, and computer science, the authors conducted separate literature searches in databases from each field. These included 1 medical-focused database (PubMed); 1 computer-focused database (ACM Digital Library); and 2 multidisciplinary full-text databases (SpringerLink and ScienceDirect) from 2 leading publishing groups, Springer and Elsevier, respectively. Our search was tailored to the review topic followed by an analysis of text words found in titles, abstracts, and keywords used in retrieved papers. The electronic search was conducted in December 2023 using keyword combinations in the title, abstract, and keywords fields to ensure comprehensive coverage. Keywords were selected and classified into 4 categories: OMC (subject of the study), recommendation (objective of the study), OHC (fields of the study), and excluded keywords—queries 1 to 4. To emphasize the recent advancements, an additional query 5 set a time limit from January 1, 2001, to November 1, 2023. The overall search strategy was 1 AND 2 AND 3 AND (NOT 4) AND 5. Table 1 presents the hierarchical search query and all keywords.

Following the keyword search, a reference list search (ie, backward reference search) and a cited reference search (ie, forward reference search) were conducted on the full-text articles that met the study selection criteria. Using the results of the backward and forward reference searches, the same study selection criteria were applied to further screen and evaluate articles. We repeated these procedures on all newly identified articles until no additional relevant articles were found.

**Table 1:** Literature search strategy.

Search number	Search	Keywords
1	Title	*(doctor OR physician OR consultation OR treatment OR e-health OR m-health OR telehealth OR remote health OR digital health OR online medical service OR web-based, health OR internet-based, health)*
2	Title	*(recommendation OR recommender OR recommending OR matching OR rating OR choosing OR selection)*
3	Title/abstract/keywords	*((Health OR Healthcare) AND (Communities OR Forums OR Platforms))*
4	Title/abstract/keywords	*(Qualitative research OR Practice)*
5	Time range	*January 1, 2011, to November 1, 2023*

### Eligibility Criteria

**Figure 2:**
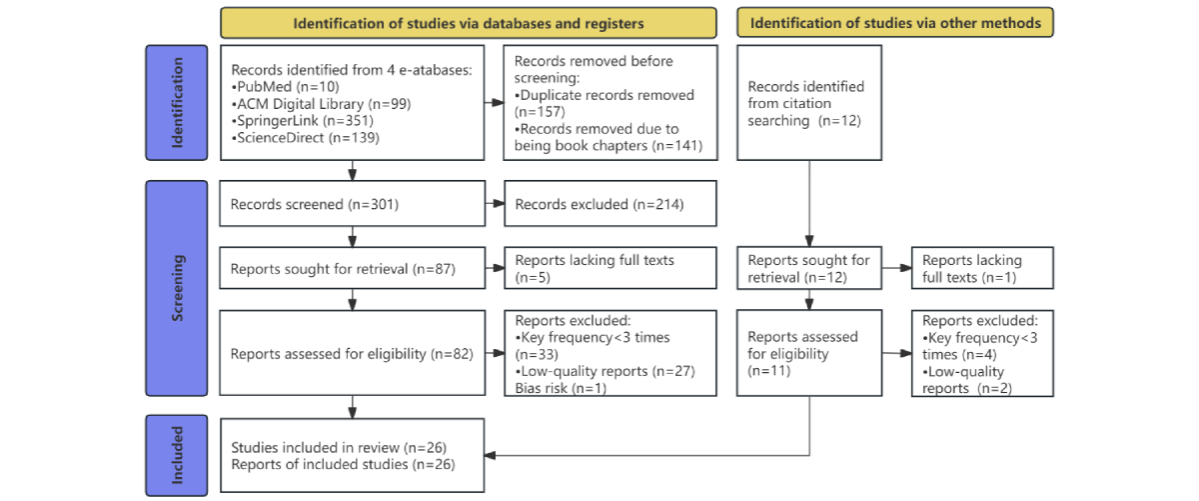
PRISMA (Preferred Reporting Items for Systematic Reviews and Meta-Analyses) diagram of the study.

The titles and abstracts of identified articles were independently screened by 2 researchers (HJ and ZM) to determine inclusion in the full review. Figure 2 illustrates the paper selection process. If either or both reviewers selected the paper for further evaluation, it was included for full assessment. Articles were considered for analysis if they met at least one of the following criteria: (1) OHC-oriented physician recommendations, (2) coding or documenting of patient preferences, (3) motivations or perceptions of physicians involved in OHCs, (4) implementation of a recommender system for medical services, and (5) recommendation acceptance and interface design in the domain of medical-related recommender systems. Disagreements were resolved with a third reviewer (WX) until consensus was reached. In addition, articles must have met the following three criteria to be considered for analysis: (1) published in peer-reviewed journals or conference proceedings, excluding research articles without detailed research designs or results; (2) written in English; and (3) published between 2011 and 2023 to align with the recent emergence of OHCs over the last decade.

### Quality Assessment

To ensure the quality of the articles, we applied a GRADE (Grading of Recommendations, Assessment, Development, and Evaluations) framework. The purpose of this initiative is to help individuals make informed decisions using evidence systematically and transparently [12]. The GRADE Evidence to Decision frameworks have been illustrated and are useful in making and using health-related recommendations and decisions [13]. They identify 4 levels of evidence for each study: very low, low, moderate, and high. GRADE criteria examine the risk of bias, imprecision, inconsistency, indirectness, and publication bias in evaluating the quality of evidence of a study [14]. In the scenario of OMC recommendation design, those studies start at high quality of evidence if they use both offline data sets and online data streams for randomized controlled experiments. In contrast, observational studies begin at a lower quality of evidence due to residual confounding. Referring to previous studies [15], only moderate and high-quality articles were selected to avoid low-quality articles.

## Results

### Search Results

The electronic database search yielded 599 studies, with an additional 12 studies identified from Google Scholar through reference list and cited reference searches for each obtained study. After removing book chapters and deduplicating entries, 49.3% (301/611) of the studies remained. The review process involved excluding 71.1% (214/301) of ineligible studies after screening titles and abstracts as they did not meet the criteria specific to the theme of physician recommendations, the OMC domain, peer-reviewed sources, or English language. This left 87 studies for full-text review, of which 5 (6%) reports were found to have no full texts and were subsequently removed. The full texts of the remaining 94% (82/87) of the studies were assessed for bias risks and qualitatively analyzed. Ultimately, 21 high-quality studies with adequate outcome data were selected for quantitative analysis. In addition, 5 studies were identified through citation searching and included in the quantitative analysis.

### Study Characteristics

Table 2 summarizes the features and discoveries of each of the 26 studies. The number of publications increased over time. This indicates that this field is receiving more and more attention from scholars and practitioners due to the global prosperity of OHCs. This review comprised studies from 6 countries across Asia, Europe, and North America, most of which were low-income countries. The largest source of articles was China, followed by Portugal and India. This reflects that, in countries with less developed offline health networks, online medical services are of particular benefit, as illustrated by the growth of OMCs in China. There were various recommendation algorithms used: analytic hierarchy process, CF, content-based filtering, decision tree, neural network, matrix factorization, and regression analysis. Although research has been conducted using a variety of methods, CF, matrix factorization, and analytic hierarchy process are the top 3 most commonly used ones, with 38% (10/26) of the studies involving their use entirely or in part. However, over the last 5 years, the research methods of graph-based deep learning have become increasingly popular. The most widely used method of data engineering in the field is text analysis, such as latent Dirichlet allocation (LDA) and word2vec, followed by knowledge graphs (KGs).

Of the 26 studies included, 13 (50%) used data directly from OHCs as their source, whereas 7 (27%) used data indirectly from the official websites of hospitals or health care centers. Depending on the sources, the data sets could be classified as online or offline. There are numerous OHCs that produce massive amounts of heterogeneous, multimodal, and high-dimensional raw data continuously [16,17]. Online data generated by these OHCs support medical diagnosis and decision-making. Offline data are usually collected from various medical institutions and typically stored in health care information systems. They are exported once permission has been granted [18-22]. In addition, many studies collected primary data through questionnaires directed at patients or physicians [23]. Depending on the objects described, the data sets could be classified as patient, physician, and institution data, as shown in

Table 3. The data quality of physician profiles, such as educational background, professional experience, disciplines, and expertise, was high and well defined. The patients were OHC users and service consumers, but they were nonexperts or laypersons in the medical field who usually chat on the web without any restrictions or limitations, which results in poor-quality data from their consultations. This makes data processing and feature extraction quite complicated and challenging.

**Table 2:** The bibliographic characteristics of the included studies.

Study	Date	Country	Study aim	Method	Data sources
Huang et al [18]	December 2012	China	Using patient preferences and physician performance to recommend doctors	CF^a^ and AHP^b^	Official appointment platform for the Shanghai Medical League
Jiang and Xu [24]	December 2014	China	Combining the relevance and quality of doctors in an integrated recommender system	Semantic similarity computing and AHP	OHCs^c^: HaoDF, XYWY, Ask39, and 51daifu
Gong et al [19]	September 2015	China	Using medical social networks and a medical data set to recommend doctors	Time-constraint probability factor graph and random walk with restart	Clinic experiments at the Chinese Academy of Sciences
Narducci et al [25]	May 2015	Italy	Delivering a semantic recommender system based on social networks	Similarity computing and CF	—^d^
Guo et al [26]	July 2016	China	Identifying KOLs^e^ using health care data mining for any specific disease	Unsupervised aggregation approach	Medical journal papers
Zhang et al [27]	January 2017	China and United States	Using topic modeling and emotional offset to recommend doctors	Matrix factorization, LDA^f^, and sentiment analysis	Yelp
Sridevi and Rajeshwara [28]	August 2018	India	A personalized physician recommender	Similarity computation and combined ratings	—
Han et al [20]	October 2018	Portugal	Establishing a mechanism for matching patients with family doctors	Hybrid matrix factorization and latent representation	Consultation histories of a leading European health care provider
Waqar et al [23]	January 2019	Pakistan	Combining content-based and collaborative and demographic filtering to create a hybrid physician recommender	Content-based filtering, CF, similarity measure, and AHP	Survey data from 3 hospitals in Islamabad, Pakistan
Pan et al [29]	January 2019	China	Personalizing physician selections based on patient preferences and illness conditions	Dynamic assortment planning and upper confidence bound	Simulation data
Xu et al [30]	June 2019	China	Recommendations based on doctors’ reputation scores and similarities with patients’ demands	Truth discovery, modified Paillier cryptosystem, and Dirichlet distribution	Simulation data
Ye et al [31]	August 2019	China	Picking doctors using signaling theory with patient needs	Binary long short-term memory, LDA, regression, and AHP	OHCs: HaoDF and XYWY
Yang et al [32]	February 2020	China	Enhancing physician recommendations based on patient preferences	Intuitionistic fuzzy sets and Bonferroni mean	OHC: HaoDF
Wen et al [33]	April 2020	China	Providing real-time personalized recommendations by optimizing limited physician resources	Adjusted exponential inventory balancing	Simulation data
Mondal et al [21]	October 2020	India	Modeling patient-physician relationships to recommend doctors	Multilayer graph data model	Records from health centers and hospitals
Yan et al [34]	October 2020	China	Fusing review text and physician information to improve medical consultation recommendations	Convolutional neural network and probabilistic matrix factorization	OHC: HaoDF
Meng and Xiong [35]	January 2021	China	To propose a hybrid physician recommendation model based on OHCs	Eigenvector, word2vec, and LDA	OHC: Chunyu
Peito and Han [36]	January 2021	Portugal	Developing a content-based matchmaking system for patients and doctors	Pretrained Poincaré embeddings and transfer learning	A data set of a European private health network
Wang et al [37]	January 2021	China	Proposing a diversity-enhanced hierarchical physician recommendation approach	Matrix factorization and heuristics	OHC: HaoDF
Ju and Zhang [38]	August 2021	China	Ontology-based recommendation of doctors based on disease text mining	Ontology and text mining	OHC: GuaHao
Yuan and Deng [4]	February 2022	China	Using knowledge graphs and deep learning to recommend doctors based on OHCs	Knowledge graph and deep learning	OHC: HaoDF
Lu et al [39]	May 2022	China	Recommending doctors through expertise learning in OHCs	Multi-head attention and pretrained BERT^g^	OHC: Chunyu
Chen et al [40]	July 2022	China	Considering patients’ risk preference in a probabilistic linguistic environment to recommend doctors	Probabilistic linguistic term set, TF-IDF^h^, and word2vec	OHC: HaoDF
Wang et al [41]	August 2022	China	Developing a model to predict patients’ preferences regarding medical consultations based on physician characteristics	LASSO^i^, multilayer perceptron, decision tree, and Shapley Additive Explanations	OHC: HaoDF
Wu et al [42]	February 2023	China	Making a decision-making method for online physician selection that considers correlation	Choquet integral, BERT, and 2-additive fuzzy measure	OHC: DXY
Valdeira et al [22]	August 2023	Portugal	Physician recommendation with implicit feedback and limited patient information	Deep extreme classification with label features	Consultations of a European private health network

^a^CF: collaborative filtering.

^b^AHP: analytic hierarchy process.

^c^OHC: online health community.

^d^Not applicable.

^e^KOL: key opinion leader.

^f^LDA: latent Dirichlet allocation.

^g^BERT: Bidirectional Encoder Representations from Transformers.

^h^TF-IDF: term frequency–inverse document frequency.

^i^LASSO: least absolute shrinkage and selection operator.

**Table 3:** The online health community data set contains various categories and features, including information related to physicians, patients, and hospitals.

Category and features			Description
**Physician profiles**			
	ID, name, age, gender, geographic location, hospital, and department	Physician’s personal information	
	Specialties, number of patients, and professional title	Professional experience and expertise	
	Academic background, research achievements, and academic titles	Academic background	
	Patient ratings, patient reviews, and patient satisfaction	Online and offline word of mouth	
	Number of popular science articles	Physicians’ willingness to engage in science popularization.	
	Historical records	Physicians’ historical consultations	
**Patient profiles**			
	ID, gender, age, and location	Patients’ personal basic information	
	Disease description and medical history	Disease information provided by the patient in advance	
	Consulting records	Records of patient consultations with physicians	
**Hospital information**			
	Hospital grade and ranking	Hospital reputation	

### Data and Feature Engineering

An accurate acquisition of features enables an effective recommendation system, and feature engineering forms the foundation of personalized recommendation systems. Data engineering begins with raw data preprocessing. Duplicate or missing values can be handled by deleting them or using average values. Semistructured data, such as the demographics of physicians or patients, need to be converted into structured data by recognizing named entities and extracting information. When analyzing unstructured data, such as physician-patient consultation records, the content may be nonstandard, repetitive, short, and straightforward. Pycorrector, a third-party open-source library developed by Python, can be used to correct some common errors in oral expression [38]. Afterward, word separation, deactivation removal, normalization, and other procedures are performed. A word separation process extracts and vectorizes text features. Considering the specificity and professional nature of the medical field, the consultation records contain many medical professional words, and synonymous disease names must be substituted (eg, the term “trisomy 21” indicates a pediatric Down syndrome disorder). To ensure that professional terms are recognized during word segmentation, it is recommended to develop a dictionary based on medical ontologies. Furthermore, medical experts can be consulted to refine the dictionary by deleting terms outside the required domain [4]. Afterward, stop words should be removed to eliminate meaningless words or characters and reduce noise. For word segmentation, the most commonly used tools are Jieba and WordNet Lemmatizer in the Natural Language Toolkit library; for removing stop words, the most commonly used lexicons include the Harbin Institute of Technology stop word list, the Baidu stop word list, and stop words in the Natural Language Toolkit library.

OMC recommendations also face data sparsity challenges. Domain specialization leads to data sparsity. An OMC is not a domain of fast-moving consumer goods but a professional service. Most people do not consult physicians regularly but rather initiate consultations only when they need one, such as when a condition arises. In most cases, patients will consult only 1 physician for a condition or disease. Once cured, they will not revisit the same physician; otherwise, they will try another physician. In other words, it is rare for a physician and patient to have multiple records of the same condition or disease. Despite OHCs having an extensive collection of physicians, most of those physicians are considered “silent” in the communities as, in most cases, patients pay attention only to those physicians who are well-known and highly regarded. It was only possible for patients to rate or write reviews for physicians they had consulted rather than for other physicians. All these factors contribute to data sparsity.

To alleviate data sparsity, either the model should be improved or more features should be mined. According to the literature [30], patients’ uncertain characteristics and preferences could be revealed through uncertainty languages, and fuzzy analysis could be used to improve recommender systems’ sparsity problem. KGs have been introduced to represent physician-patient interaction features in the physician recommendation problem, thereby alleviating data sparsity [4]. A sociosemantic approach was used to address the problem of data sparsity caused by user-based CF [43]. Son and Choi [44] used ordinal and binary ratings of experts to refine user opinions and mitigated data sparsity in hand-edited expert recommendations. Wang et al [37] proposed a matrix decomposition to handle sparse data and improve prediction accuracy.

**Figure 3:**
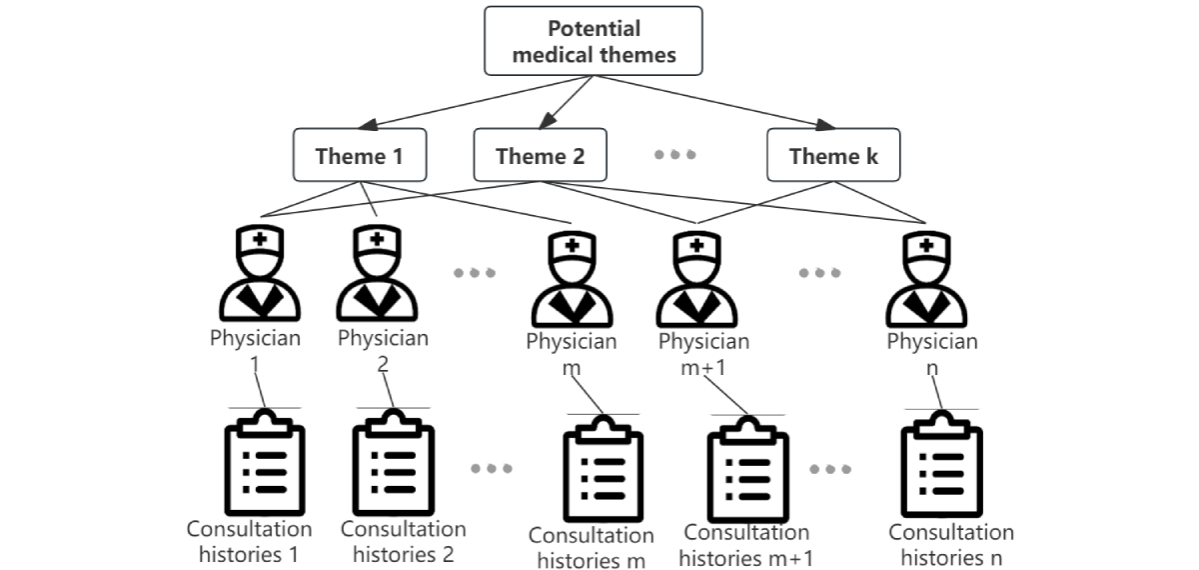
Identifying each physician’s specialty involved analyzing their historical consultation texts using medical terminology recognition and topic classification mining.

Medicine is a very specialized field of science. Often, because of cognitive limitations, patients cannot express their conditions and medical histories in consultation content, and some are unable to even express their personal needs. Using topic models, unstructured texts are analyzed for their content to retrieve, classify, cluster, summarize, and find topics that have similarities or relevance. The most common topic modeling method, LDA, uses an unsupervised probabilistic model to generate topics [31]. Typically, LDA is used to extract topics from large data sets of documents by mining potential semantic relationships between them. Meng and Xiong [35] used all physician consultations as a corpus for LDA, as shown in Figure 3, and each physician’s text-topic distribution was then used to train a model to retrieve the corresponding physician for a specific topic. Zhang et al [27] applied LDA to extract patients’ potential preferences and the characteristics of the physicians they consulted from patient reviews. LDA has some shortcomings. First, LDA lacks semantic contextual information when processing text because the commonly used bag-of-words model ignores it [34]. Second, LDA models perform poorly when text topics are too sparse to represent potential features; training LDAs tends to overfit if there are too many topics, so a fair number of topics must be selected to strike a balance between the degree of fit and simplicity. Finally, LDA models cannot handle labeled data on documents, causing uninterpretable topics to be generated. From various perspectives, scholars have proposed solutions to the aforementioned drawbacks. Ye et al [31] reduced the time complexity of LDA via Gibbs sampling and determined the optimal number of LDA topics based on the confusion level. Because the patient’s “initial inquiry” text is usually short and the corresponding topic vector representation is sparse, Liu [45] used a short text aggregation algorithm to represent the topic vector. Pan and Ni [46] used a labeled LDA model to generate probability distributions for health questions and topics and topics or words based on the text set of physicians’ answers to health questions.

Sentiment analysis identifies users’ attitudes and opinions on commodities or services from their review texts. In addition to medical topics, consultations and patient evaluations on OHCs include patient emotions and feelings. Using sentiment mining techniques, sentiment information can be extracted from text data. Text sentiment analysis can be divided into 2 main types: lexicon based and deep learning based. Sentiment dictionaries are the traditional tool for analyzing words and short texts’ sentiment tendencies [31]. These dictionaries describe not only the positive and negative sentiment attributes of words in static dictionaries but also the offsets of sentiment information of words in sentence frameworks. The China National Knowledge Infrastructure, the Information Retrieval Laboratory at Dalian University of Technology, and the Natural Language Processing and Social Humanities Computing Laboratory at Tsinghua University are 3 dictionaries commonly used for sentiment analysis of Chinese texts. On the basis of sentiment dictionaries, Zhang et al [27] used unsupervised learning methods to calculate the offset between patients’ comments and their sentiments and correct the original patient ratings. There is evidence that deep learning is superior in the analysis of long texts containing complex sentiments. To analyze positive and negative sentiments in patient reviews, Ye et al [31] used the binary long short-term memory method, which achieved better results than sentiment dictionary analysis. For sentiment polarity analysis in review texts, Wu and Sun [47] used the Bidirectional Encoder Representations from Transformers model, and for recommendation results, they applied the Wilson interval method. Due to the subjective nature of patient comments and the unreliability of sentiment ratings, sentiment mining methods have limitations. Data sources of uneven quality can also affect the accuracy of sentiment evaluations. The fuzzy analysis of the text can help address the uncertainty of text description [48]. The fuzzy analysis mainly applies fuzzy mathematical or fuzzy linguistic methods, which allow recommender systems to express uncertainty and obtain personalized features from patient comments. Intuitionistic fuzzy numbers serve as effective tools for dealing with fuzzy information (ie, describing the degree of neutrality in uncertain situations). Yang et al [32] converted raw data into intuitionistic fuzzy numbers to describe uncertainty information by combining the patient’s disease description with comments. Xu et al [48] examined data based on hesitant fuzzy language multi-criteria preference analysis to enhance patient preferences for physician recommendations.

### Recommendation Algorithms

#### Overview

OMCs recommend a service with suitable physicians according to the patients’ needs, an application scenario differing from that of item recommendation in e-commerce and rather resembling expert discovery in online question and answer (Q&A) communities or academic peer review. These recommendations have one thing in common: the recommended subject is not a product but rather a human, a competent and knowledgeable professional. A physician’s expertise can be inferred from their educational and professional background as well as historical consultations, similar to the history of expert responses in Q&A communities or the list of academic papers of a scholar. Patient comments and ratings for a physician are similar to the number of likes for a Q&A expert or citations of a scholar. It can be compared to assigning a competent academic reviewer to a new topic, finding a suitable expert to answer a new question, or recommending an appropriate physician based on graphic descriptions of the patient’s consultation.

#### Knowledge-Oriented Recommendations

Knowledge-intensive service recommendations are determined by matching large amounts of textual information between patients’ inquiries and physicians’ skill sets. In general, the better the information match, the more likely it is that the service recommendation will be successful. As part of content-based recommendations, physicians’ backgrounds and historical data are gathered, and textual topic techniques are used to mine their expertise, such as LDA, probabilistic latent semantic analysis, and so forth. Pan and Ni [46] modeled the textual topics of historical consultations and physician responses under each section, mined physician expertise using labeled LDA, and completed physician recommendations based on candidate physician expertise and pending inquiries.

**Figure 4:**
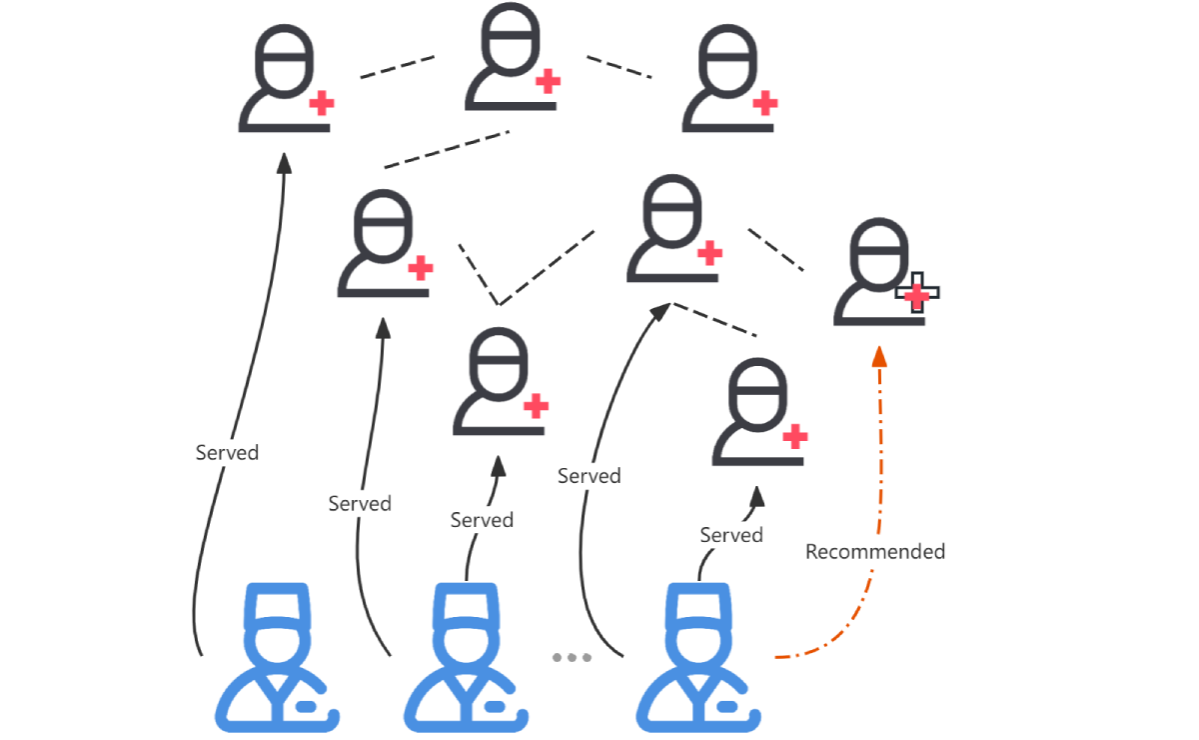
Social network–based recommendations leverage patient-friend relationships to recommend a physician to a new patient with a similar condition, aiming to gain the trust of the new patient.

Social network–based expert recommendations have grown in popularity and are derived from a classical algorithm of information retrieval (ie, PageRank). For expert recommendations, Wang et al [49] proposed a convolutional neural network for answering online expert questions that effectively reduces waiting time for the questioner and improves the quality of the answer. To alleviate the cold-start problem for new-coming patients, physician recommendation–related studies should consider patients with similar conditions in the OHC who exchange information and provide emotional support, as illustrated in Figure 4. Recently, expert recommendation research has increasingly incorporated integrated models that combine features such as social networks and knowledge content. Xu et al [50] proposed a scholarly recommendation framework that integrates social network analysis and conceptual semantic analysis in 2 dimensions: social relationships among scholars and information about their expertise. Yang et al [51] used information about research relevance, personal social networks, and institutional connections to identify the most appropriate experts for collaboration on research. Xu et al [52] proposed a methodology for a collaborative recommendation that integrates expert expertise and social information in a complex heterogeneous network using heterogeneous network mining. It identifies valuable meta-paths through information gain and uses regularized optimization to generate personalized recommendations tailored to each scholar’s needs. Different recommendation algorithms have different strengths in comparison. Expert recommendations based on knowledge content are better suited for use in enterprises with high levels of information quality and clearly defined knowledge hierarchies. Information quality in OHCs is significantly lower than that in general organizations, and expert recommendations are greatly influenced by the structure of social networks [53]. Both of these features are present in the OMC service recommendations studied in this review.

**Figure 5:**
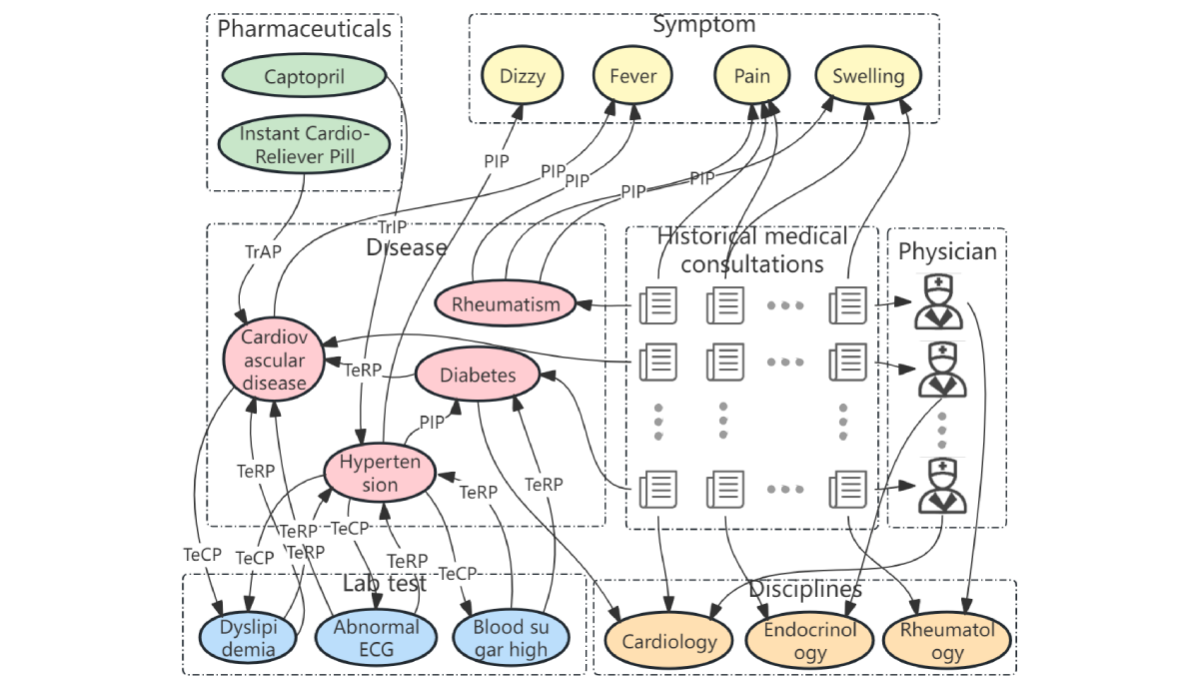
Constructing a physicians’ knowledge graph—an example in cardiovascular medicine. The knowledge graph comprises various medical entity nodes, such as specialized disciplines, diseases, symptoms, and pharmaceuticals. These entities are extracted from the historical consultation records of physicians. ECG: electrocardiogram; PIP: medical problem indicates or reveals aspects of another medical problem; TeCP: test given to investigate a medical problem; TeRP: test reveals a medical problem; TrAP: treatment administered for medical problem, but outcome is not mentioned in the sentence; TrIP: treatment improves or cures medical problem.

A KG is a structured semantic knowledge base that integrates heterogeneous information from multiple sources and represents rich entity relationships using complex networks, which facilitates the storage, processing, and communication of complex real-world knowledge. Medicine is a specialized scientific field, and vector representations of KGs enable algorithms to obtain embeddings of concepts, class hierarchies, entities, and relationships and, in turn, graph structures, paths, and subgraphs. Algorithms can achieve logical reasoning in vector space with the help of ontology embedding and rule learning. For the OHC platform to be credible, physicians must provide their real names, educational backgrounds, professional experience, and expertise so that their profiles can be verified. Using document clustering analysis, LDA topic segmentation, and feature extraction from physician historical consultations, a KG describing physician specialty and expertise can be constructed. Yuan and Deng [4] produced a more accurate and interpretable recommendation scheme based on the KG to overcome the problem of sparse data. It is common practice for existing studies to extract entities based on physician historical consultations; however, these data alone are not sufficient to represent physician professional specialties. For example, if an otolaryngologist has only received consultations related to the ear and nose for various reasons, then the system only measures their expertise in the ear and nose. However, in practice, they also have excellent expertise in laryngology, which the system cannot calculate. An appropriate recommendation system should be designed to recognize the differences between specific diseases and the expertise of various physicians within the same department. As shown in Figure 5, the original scope of historical consultations should be extended to include new entity nodes such as specialized disciplines, physicians, and consultations. To optimize the network structure of the KG, we should analyze the semantic connotation of keywords, determine the semantic similarity between consulting cases and their attribution to specialized disciplines, and examine the professional areas of physicians and their evolution trends.

KG-based physician recommendations are a new trend in OMC service recommendations. By using logistic regression, plain Bayesian classification, and noise-immune gate Bayesian networks, Rotmensch et al [54] constructed a KG, and from the parameter training, a disease-symptom topological relationship graph was generated. Liu [45] used the k-means algorithm to cluster physicians and generalized goodness-of-fit metrics to evaluate and adjust the clustering results. By comparing the patient’s consultation content with the physician clustering center and the individual physician information in each category, a physician category and physician object that are more closely matched could be recommended. Xu et al [52] proposed a collaborative recommendation method for scholars based on heterogeneous network mining, combining expert expertise with social information, identifying valuable meta-paths through information gain, and providing personalized recommendations for each scholar through canonical optimization. On the basis of the similarity of consultation texts, Meng and Xiong [35] constructed a co-occurrence label network of physicians and calculated the centrality of the feature vector to recommend the most important physicians. Gong et al [19] proposed a hybrid multilayer architecture, iBole, of physician recommendations, mining physician-patient relationships using a time-constrained probabilistic factor graph model and recommending physicians based on random wandering. KG-based physician recommendations also have drawbacks. The OMC service faces more complicated application scenarios involving multiple entities and interentity relationships that reflect a physician’s knowledge or disease-symptom connection. It is difficult to integrate different attributes and relationships between attributes in traditional recommendation methods, and it is nearly impossible to visualize the relationship between each knowledge attribute and physicians. KG-based OMC service recommendations should use multisource heterogeneous information to mine physicians’ comprehensive expertise, take their profiles as basic professional descriptions, mine all their published articles using text semantics, and then combine their historical consultations with multimodal data to extract features using multimodal mining and LDA topic segmentation.

#### Interpretable Recommendations

As medicine is such a specialized field of science, recommendations must be interpreted according to the patients’ cognitive capacity. It is difficult for patients to make autonomous judgments about the recommendations using their knowledge because they lack theories and relevant experience. Most of the existing research on recommendation systems is devoted to the professional accuracy of recommendation results. It casually ignores the interpretability of recommendation schemes and the lack of transparency in the system computation process [4]. In other words, the recommendation process and logic are not adequately explained to patients considering their cognitive capabilities. It is very critical for the recommendation system to be interpretable as it directly correlates with the level of trust of patients [55]. To provide patients with a reference for decision-making, we believe that a good recommendation system for OMC services must incorporate an interpretable and user-friendly recommendation algorithm. As a result, patient acceptance and recognition of the recommendation results will be enhanced, which will ultimately result in a higher acceptance rate of the recommended solution of the system. As a result of their limited cognitive abilities, many patients, in addition to not judging the recommendations, struggle to make their inquiries clear and complete, and in a few cases, they even cannot accurately articulate their personal needs. As an alternative to solving such difficult problems, multimodal data mining techniques may be considered, such as multimodal graphical topic modeling for patient description and consultation needs. Not only can key information from patient consultations be explored and labels can be extracted, but it is also possible to avoid creating too sparse input text variables by avoiding personalized verbal expressions and symptoms. Machine learning algorithms can easily process clustered documents when they are converted into vector distributions.

Recommendation algorithms can be interpreted in light of the rich semantic connections between physicians and patients in the KG [4]. Some studies have demonstrated that interpretable recommendation algorithms based on KGs enhance the level of patient trust. Using KG-based disease diagnosis algorithms, Wu and Sun [47] obtained initial disease alternative sets by querying the KG and using the KG embedding model; the KG-structured information was used to enrich the disease alternative set, enhancing the recommendation accuracy and facilitating the recommendation of potential diseases to the user. To identify the different roles of physician-patient interaction characteristics and individual physician characteristics in physician recommendations, Yuan and Deng [4] developed a deep learning model that can provide accurate and interpretable physician recommendation information by combining layer-by-layer association propagation techniques with deep neural networks. Considering the accuracy, diversity, and interpretability of KG-based recommendations resulting from information such as rich semantic relationships and item links within a network, we propose that interpretable recommendations should be built based on KG path inferences. The algorithm should adopt a knowledge-aware path recurrent network model, which generates path representations by combining the semantics of entities and relations, reasoning by using sequential dependency in paths to infer interaction between users and items, and incorporating a weighted pool into the process of inferring user preferences to differentiate between different contributions from different paths to provide interpretable recommendations.

### Evaluation

Physician recommendations can be evaluated online or offline. Online evaluation involves measuring the effectiveness of the recommendation system by obtaining the target users’ evaluation of the recommended object, namely, the rating of the recommended physician by patients. Guo et al [26] asked 3 faculty members and 3 graduate students with medical backgrounds to judge candidate physicians based on their perception of their professional activities and reputation and use the mean of the ratings to rank them. Ye et al [31] recruited 18 students with experience in helping relatives choose a physician to consult on the web and asked them to assess the relevance of the physician in response to a given consultation question. Wu and Sun [47] used a questionnaire to assess the accuracy of a physician’s recommendation and validate the proposed recommendation algorithm, including whether the respondents had had a particular disease, had been treated in the area, and had approved of the physician. An online evaluation has several shortcomings, including a high implementation cost and the difficulty of excluding the characteristics of the group surveyed as well as personal subjective factors from the results. An offline evaluation involves feeding training set data into the system for training the recommendation model and calculating the recommendation results based on test data to measure the performance of the recommendation system. In most cases, machine learning models are trained through supervised learning, which means that the predicted output of the recommendation model is compared with the true value, and based on the difference, model training methods can be altered and parameters can be adjusted to facilitate the continuous optimization of the model [53]. There are different measurement criteria for the difference between the predicted output of the model and the true value. Offline evaluations are predominantly based on accuracy, which includes classification accuracy, prediction accuracy, and ranking accuracy.

The diversity and coverage of recommended physicians have also been used to evaluate the performance of recommendation algorithms. According to the literature [32], recommending only similar physicians results in a limited choice for patients and an imbalance in physician use. Patients will be more likely to engage with the recommender system if there is more diversity of recommended physicians. A measure of coverage refers to the proportion of recommended physicians to all physicians [56]. A low level of coverage indicates that a limited number of physicians are available to patients. Patients are likely to be less satisfied with a recommender system if the candidate pool is limited. However, diversity and coverage metrics are not currently heavily used for evaluating physician recommendation systems. Physician recommendations differ from traditional e-commerce recommendations in some respects. Patients should be recommended physicians with similar expertise or experience that matches their disease conditions rather than a greater variety and number of physicians. Increasing the diversity and coverage of physician recommendations is unfavorable to patient outcomes, thereby affecting the application of these 2 metrics in physician recommendations.

## Discussion

### Principal Findings

**Figure 6:**
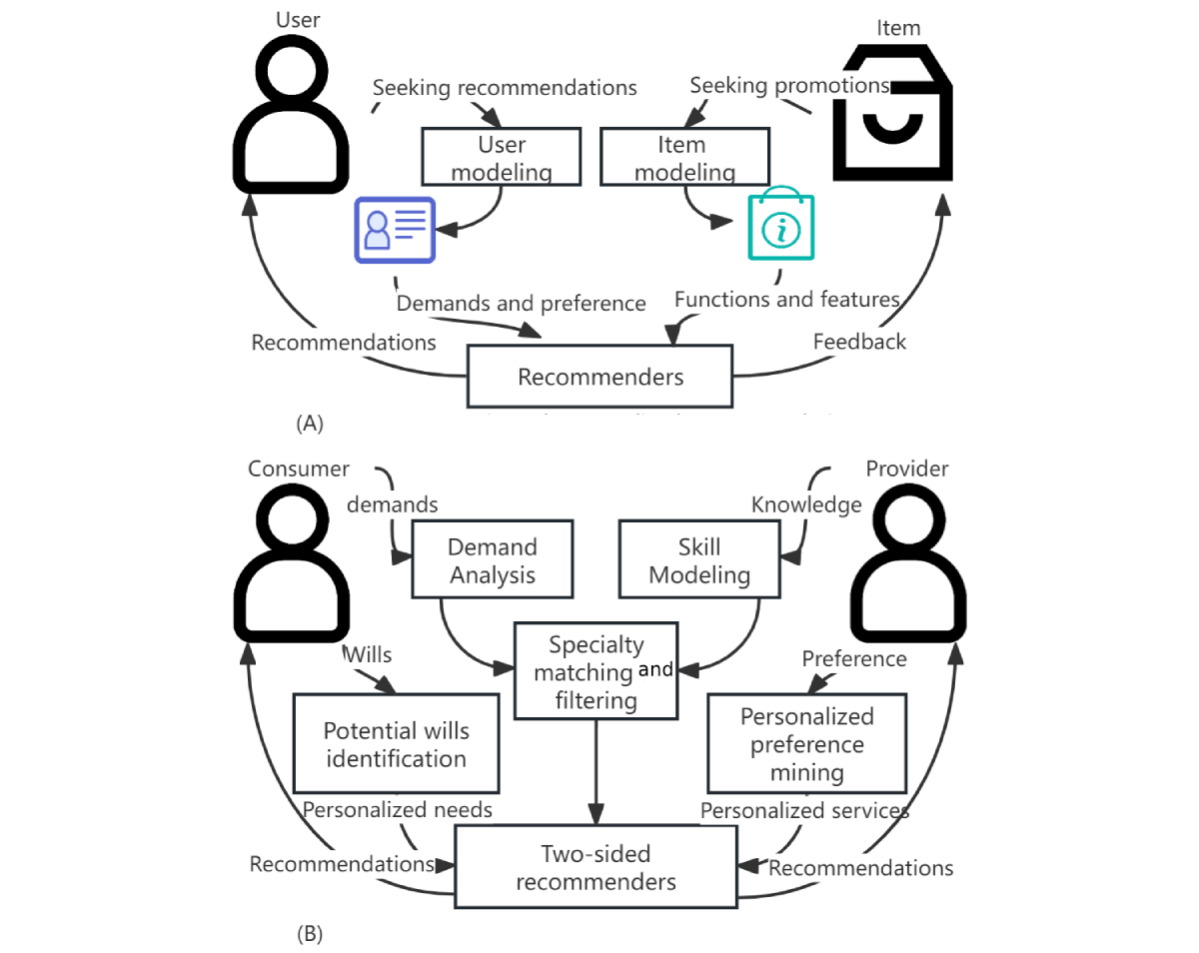
The distinction between commerce-oriented and service-oriented recommendations. Commerce-oriented recommendations focus solely on user preferences, disregarding item recommendation limits, whereas service-oriented recommendations must consider the needs and preferences of both parties involved as well as the providers’ capacity constraints. (A) e-Commerce oriented personalized recommendation system; (B) e-Service oriented personalized recommendation system.

Personalized recommendation studies have previously focused on commodity recommendations based on “users versus items” and rarely considered service recommendations based on “users versus users.” This paper focused on human carriers who deliver OMC services, particularly when recommending professional services. The OMC service represents a new form of e-business under the knowledge economy as well as a new direction for the development of e-services. Figure 6 shows that a knowledge service–oriented recommendation differs from a traditional commodity-oriented recommendation from a system thinking perspective.

An independent service-oriented recommendation system requires a novel theoretical framework and its key techniques. Table 4 illustrates the comparison between e-commerce and e-service recommendations. First, earlier studies only considered the interests and preferences of the user, not the feelings of the providers recommended; the adoption of an OMC recommendation depends not only on the opinion of the consumer but also on the preference of the service provider. It is impossible to achieve even the so-called “best” recommendation scheme by focusing only on the needs of consumers and ignoring the individual preferences of service providers. Moreover, as the physician is more aware than the patient, they should have a higher priority in terms of decision-making [57]. Existing personalized recommendation systems have obvious flaws and weaknesses both theoretically and algorithmically even when designed specifically for consulting services. Although the recommended subjects in some expert recommendation system research, such as thesis review, project approval, and other scenarios, are also humans, the recommendation algorithm still focuses on the personalized characteristics of the demand side, analyzing only the professional skills of the experts rather than considering their preferences. These experts are just “tool men.” In the case of e-service recommendation applications such as OMCs, such a research perspective and research conclusions are not applicable. Due to the existence of intrinsic and extrinsic needs of 2-sided users, it is apparent that a new paradigm of personalized recommendation research must be based on a service-oriented approach.

The professional characteristics of the service require that the system provide consumers with explainable recommendations according to their cognitive levels. Medical diagnosis and treatment is a very specialized field. Most patients do not have a very clear understanding of it. The model should be capable of explaining the recommendation schemes so that patients can make informed decisions [4]. In the case of e-commerce–oriented recommendations, interpretability is not required as users understand the utility of the items and what they desire. Thus, the system simply needs to fully exploit the hidden needs and interests of users. Algorithms focus primarily on collecting users’ side information to identify their potential needs and respond to their individualized preferences [58]. Due to the consideration of medical privacy in the OMC scenario, the system is unable to extract patients’ hidden medical histories or other information from their historical treatment records [30]. Furthermore, patients generally lack medical knowledge and are unable to make independent judgments about the recommended results. Having interpretable algorithms improves not only the transparency of the recommendations but also the trust and acceptance of patients, which improves postevent satisfaction with physicians [4].

OMCs’ particularity is also reflected in its knowledge- and labor-intensive nature. OMCs are professional consultations and intellectually demanding services that involve bilateral interactions between physicians and patients [59], so physician workload must be carefully considered. Traditional e-commerce–oriented recommendation algorithms typically produce “popular products” or “superstars,” which do not consider the overwork of physicians. In reality, it is impossible to achieve an overloaded recommender scheme regardless of how well the patient’s condition matches the physician’s specialty. A few studies have addressed the “diversity” or “coverage” of recommendations; however, they only increase the total number of item types without considering the frequency of recommendations for a single item. Whenever a human-based service recommendation system is used, the workload problem must be considered, yet it has rarely been taken into account in previous studies.

Data about users are not always valuable. Whether user reviews contribute to the formulation of recommendations is also a difference between OMC scenarios and those of other applications. Several previous studies have attempted to obtain useful information from patient reviews, but these efforts have proven unsuccessful [60,61]. In general, patients are attracted to “popular” physicians with many positive reviews and few moderate and poor reviews, whereas young or unknown physicians are underrepresented, with few respondents and a lack of adequate review data. In total, 3 factors contribute to this phenomenon: patients are unprofessional, physicians are uncooperative, and evaluation of services is difficult. The first challenge is that patients are incapable of evaluating the effectiveness of professional services, and no significant correlation has been found between the online reviews of patients and their clinical outcomes [3]. Second, physicians will vigorously resist unprofessional, emotional, and malicious reviews that can harm their professional reputation [62] and may even “vote with their feet” to force the platform to block complaints. Third, the success of OMC services is dependent not only on physicians’ professionalism but also on patients’ perceptions and expectations. In addition, the patient's experience also depends on whether the medical institution where the doctor works can provide advanced medical equipment and a convenient medical environment. Even the ease of use, stability, and privacy security of OHC platforms may have an impact on patients’ evaluations [63]. Until it has been established what techniques and methods are being used to extract key elements from subjective, ambiguous, and complex patient reviews, e-service–oriented personalized recommendation systems should be cautious about using comments and ratings.

**Table 4:** Comparison of commodity-oriented versus service-oriented personalized recommendations.

	e-Commerce oriented	e-Service oriented
Components	Users vs commodities	Users vs users
Recommended items	Commodities	Services
Decision makers	Only users	2-sided users
Personal preferences	Only users	Both patients and physicians
Workload	—^a^	Physicians
Reviews and ratings	Important features	Useful but needs caution
Interpretability	Optional	Required

^a^Not applicable.

### 2-Sided Preferences

#### Overview

Personalized recommendations are based on user preferences, and acquiring accurate user preferences is key to ensuring their quality [29,32]. In contrast to other recommendations, OMC recommendations need to consider the preferences of both consumers and providers as an OHC is a 2-sided market constituted by both patients and physicians, each with independent and stable preferences. Physician preferences have regrettably been ignored in previous recommendation systems, which has resulted in infeasible recommendations. Malgonde et al [5] proposed a 2-sided recommendation framework for digital platforms to mitigate user emergence as a commercially complex adaptive system with differential and evolving goals, preferences, and constraints for both sides of a 2-sided market. The patients’ personal preferences influence their selection behavior and, thus, their satisfaction with the recommendations [23,33,38]. In turn, the physicians’ preferences influence their willingness to receive consultations, and in turn, the physicians’ onboarding and retention determine the continuity and development of the OHC [64]. Due to the differences in scale and quality of data between the 2 types of users, patients and physicians should have independent approaches to the extraction of features and the mining of behavioral patterns.

#### Patient Preferences

##### Overview

Patients’ preferences and needs have been relatively adequately explored in existing studies on physician recommendations. As shown in Table 5, when choosing a physician, patients typically consider the physician’s disciplinary background, professional competence, and institutional reputation, as well as other factors such as distance, cost, and follow-up care. To provide patients with personalized recommendations, Pan et al [29] proposed a user preference–learning algorithm to learn patient preferences. Jiang and Xu [24] proposed an integrated recommendation method that uses hierarchical analysis to screen candidate physicians based on 3 dimensions: semantic matching of physician-patient professional texts, objective evaluation of physician authority, and subjective evaluation of physician online word of mouth. Ye et al [65] used SPSS to screen patient decision factors and recommend physicians based on their composite scores. Wang et al [37] even directly used the number of visits as an important determining factor for how patients viewed the standard of care provided by physicians. Xu et al [30] investigated the privacy issues of patients and provided a multi-indicator group decision–ranking system of physicians.

**Table 5:** Various factors influencing patients’ choice of physician. The existing literature examines expertise, reputation, communication skills, location convenience, appointment availability, insurance acceptance, cost, recommendations, online reviews, and cultural and language preferences.

Study	Reputation								Service (experience)				Affordability (costs)				Others (cares)				
	Affiliation		Reputation			Word of mouth															
	Education	Organization		Position or title	Achievements		Online ratings	User evaluation		Expertise	Practices	Histories		Distance	Expenses	Following costs		Privacy	Discrimination		
Jiang and Xu [24]	✓	✓		✓	✓		✓	✓		✓		✓									
Liu et al [66]		✓		✓			✓														
Deng et al [67]				✓				✓			✓										
Li et al [68]					✓		✓	✓			✓										
Li et al [69]		✓		✓			✓				✓										
Li and Hubner [70]							✓														
Xu et al [30]					✓			✓				✓									
Xu et al [52]										✓		✓						✓			
Gong et al [71]		✓		✓			✓	✓			✓								✓		
Ju and Zhang [38]							✓					✓		✓							
Wang et al [37]				✓						✓	✓	✓				✓					
Yuan and Deng [4]		✓						✓		✓		✓						✓			

##### Reputation

“Worshipping famous physicians” has become a very common phenomenon among patients. No matter the severity of the patient’s disease, most patients prefer senior physicians from bigger institutions and more reputable practices [37]. The reputation of a physician is one of their most valuable attributes and plays an important role in patients’ decision-making process [67]. Generally, physician reputation can be divided into 2 categories: offline and online reputation [66]. The former is determined by the hospital’s rank, academic title, professional level, the number of years in the field, and the popularity of the physician, and the latter depends on patient evaluations and ratings as well as the number of votes received, acknowledgment letters, online gifts, and other factors. Patients’ cult of famous physicians is largely based on physicians’ offline reputations. Liu et al [66] found that the ranking of the hospital and the title of the physician had a direct impact on patients’ choices. The higher the title and ranking, the more popular the individual was. Deng et al [67] also concluded that the title of the physician had a significant impact on the choice of the patient. Patients favored the chief or deputy chief physician over the regular resident physician. In addition, offline reputation can moderate the impact of online reviews on patient choice. Li et al [69] demonstrated that hospital rank and physician professional credentials negatively moderate the effect of physician online ratings and activity on patient choice. Huang et al [72] revealed that a physician’s high title negatively moderated the effect on physician service ratings while positively moderating the number of service reviews. Word of mouth in OHCs determines physicians’ online reputation. The experiences of previous patients, reviews, and recommendations are important decision-making aids for newcomers. Deng et al [67] revealed that the number of views and votes received on physicians’ home pages positively influenced patients’ choice of physician. Gong et al [71] examined the impact of online reviews and online ratings of physicians on patient decisions from the perspective of trust theory. Li et al [69] found that positive physician reviews were positively related to a patient’s choice of physician, whereas negative physician reviews played the opposite role, and that negative reviews had a greater impact on a patient’s choice of a physician than positive reviews. Li and Hubner [70] demonstrated that patients preferred physicians with higher technical skills over those with higher interpersonal skills based on the different dimensions of physician ratings.

##### Serviceability

With regard to social exchange theory, physicians’ participation in OHCs is a social exchange behavior, and services such as publishing scientific articles, providing OMC services, and offering appointment registration can bring physicians financial and social rewards [6,72]. The quality of a physician’s services is reflected in patients’ online ratings and postevaluations, which in turn influence the decision to choose a physician made by potential patients in the future. Physician service quality in OHCs can be measured by the level of platform activity, engagement, responsiveness, and frequency of updating popular articles. Deng et al [67] asserted that physicians’ behaviors, such as regular updating of medical information, publication of scientific articles, and answering patients’ questions, can enhance their community reputation, which in turn can attract more patients. Gong et al [71] noted that updating physicians’ information frequently and providing quality online services were critical to building trust between physicians and patients. Using the number of physician publications of popular articles in OHCs, Li et al [69] found that physician activeness was positively associated with patient selection.

##### Affordability

It is also important for patients to consider the time and financial expense of visiting their physician when selecting a physician, preferring an appointment time and location that is convenient for them as well as cost-effective treatment options [29,32]. One of the factors that patients consider when choosing a physician is the location of the physician. Typically, patients consult on the web before consulting offline, and the location of the OMC-receiving physician is related to the convenience of future offline consultations. Ju and Zhang [38] considered the location of the patient to improve the convenience of combining online consultation with offline treatment. Deveugele et al [73] analyzed questionnaire data from 6 European countries and studied video recordings of consultations and found that the location of a physician’s hospital affected the length of the online consultation. Compared to geographical location, consultation costs have relatively little impact on patients’ choice of OMC services. Khairat et al [74] reported that costs were one of the primary factors determining patients’ choice between mobile health and telemedicine. Fletcher et al [75] also argued that the cost of providing mental health treatment via video at home was significantly lower than the cost of providing in-person care assuming that patients can make use of existing personal technology.

##### Others

The personal characteristics of a physician, such as their appearance and gender, can also influence patients’ choices. Ouyang and Wang [76] found that a serious and stable physician appearance image contributes to patients’ trust in physicians, which in turn influences their medical choices. In addition, patients have some stereotypes about physicians’ gender. The gender difference in physicians also extends to the distinction between different departments and medical specialties. Bertakis [77] found that male and female physicians practice in different ways, with female physicians providing more psychological counseling and preventive services and male physicians focusing more on technical practices such as physical examination. A physician’s gender also influences patient choice. Gong et al [71] found that physician gender influenced physician ratings and patient choice and that patient choice was enhanced when the physician was male.

#### Physician Preferences

##### Overview

**Figure 7:**
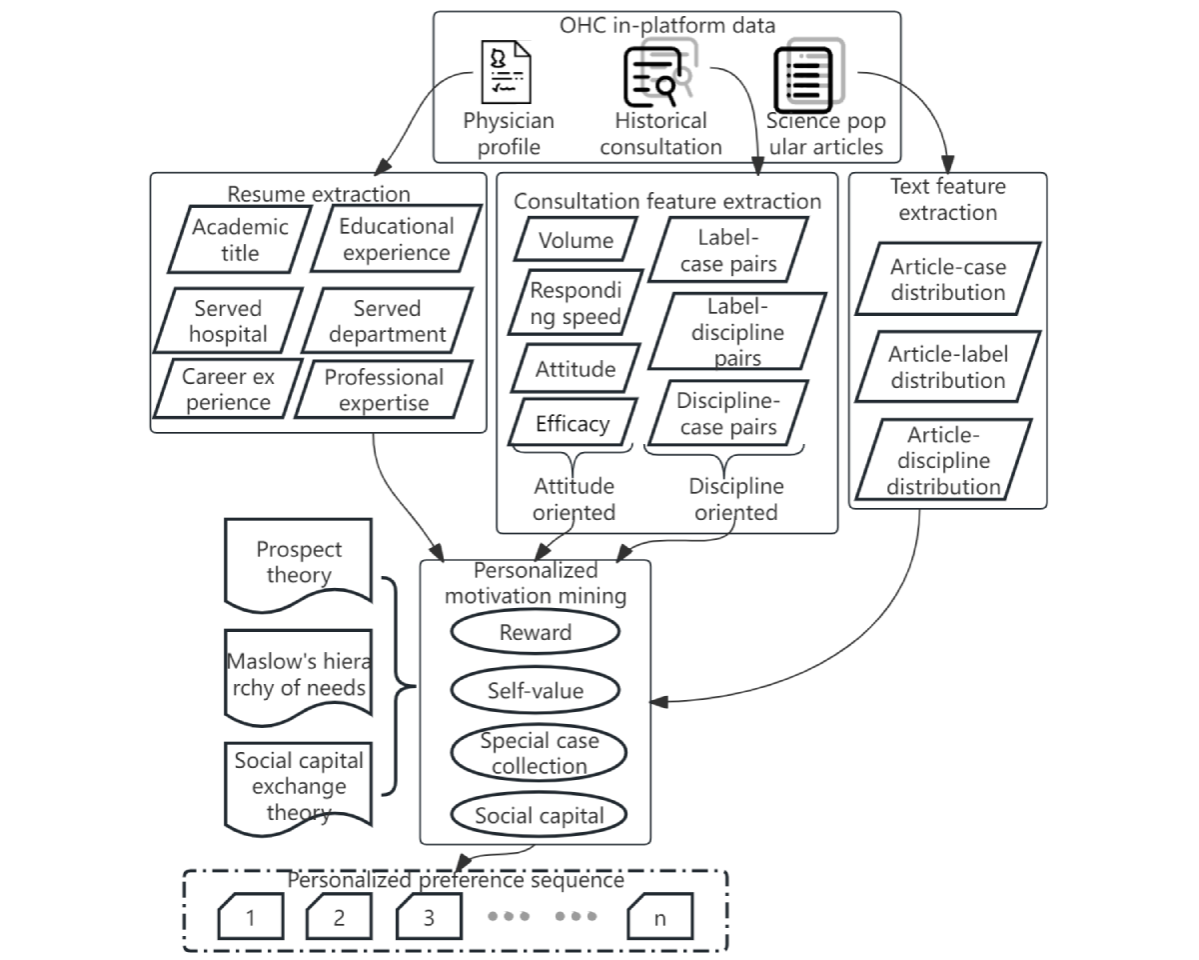
Mining physician motivations and personalized preferences. The collected data, encompassing academic titles, educational background, career experience, research accomplishments, and consultation case characteristics, inform a multidimensional preference index system. This system addresses material, career, and social capital motivations, enhancing recommendation adoption rates and personalized physician recommendations. OHC: online health community.

Continual physician involvement is crucial to the survival, growth, and prosperity of OHCs [64]. Although patients are consumers of OHCs and physicians are merely providers, the latter are of greater significance and influence. Patients who participate in OHCs seek out famous physicians, and existing OHCs are essentially physician-driven organizations [57]. In comparison with their counterparts, physicians possess a higher level of cognition and more logical behavior. There is a relatively large amount of data on physicians in current OHCs. By mining behavioral data, it is possible to gain a better understanding of their motivations and expectations. Unfortunately, most previous studies have been primarily concerned with physicians’ fitness from a professional perspective rather than with their willingness and preferences from a drive and reward perspective. Current paradigms of research, which ignore the individualized preferences of the recommended population, are not adequate to meet the growing need for human-based, knowledge-based service recommendations. According to physician motivation theory, we propose a research paradigm to examine how perceptions of personal benefits and costs, satisfaction with individual needs, and cultural differences influence physicians’ OMC decisions. Few studies have examined physicians’ preferences, and more have discussed physicians’ motivation to participate, which influences physicians’ performance in OHCs. Physicians who join OHCs and provide OMC services face both costs and rewards [6]. A rational decision is based on weighing the costs and benefits. Physicians incur cognitive costs, which include fatigue, pain, and irritability generated by providing knowledge- and labor-intensive services, and implementation costs, which include time, material, and financial costs. Physicians receive a variety of rewards, including both social and economic rewards. The former describes that a physician is respected and valued by their patients for the services they provide in OHCs as well as for fulfilling their own needs and realizing their self-worth, and the latter represents that a physician receives both direct financial gains from OMCs as well as virtual gifts and bonuses from their patients. Financial and social rewards are significant factors influencing physicians’ engagement in OHCs and OMCs. Physicians’ expectations also play a role in the extent of their influence. Figure 7 illustrates how data mining of physicians who participate in OHCs and determining their motivation to participate in OMCs can be carried out. Data collected include but are not limited to academic titles, educational background, career experience, scientific research accomplishments, and case characteristics associated with their historical consultations. The objective of mining these data is to develop a multidimensional preference index system for material motivation, career motivation, and social capital motivation. This will enable us to improve the adoption rate of recommendations and promote a personalized physician recommendation system.

##### Motivation

Physicians’ motivations for joining OHCs are remarkable in their diversity. Physicians are concerned not only with financial rewards but also with career planning, professional reputation, and social capital. These considerations include the need for self-worth realization, prestige, social support, and personal branding [64,78]. The needs theory by Maslow [79] suggests that prestige contributes to self-realization. Social exchange theory also reveals that self-realization, prestige, and social support positively influence physicians’ willingness to provide online services, whereas executive costs negatively impact their willingness to do so. Using expectancy theory, Chen et al [64] found that both external motivation (eg, external rewards and expected relationships) and intrinsic motivation (ie, a sense of self-worth) positively influenced physicians’ willingness to provide consultation services, whereas consultation time, as a major cost, negatively moderated the relationship between physicians’ willingness to serve and their behavior. Zhou et al [80] combined mental health–related OHCs with motivation theory and demonstrated that both intrinsic (technical competence) and extrinsic (network reputation and financial rewards) motivations positively influenced psychologists’ voluntary behaviors. Yang et al [81] suggested that physicians’ contributions to OHCs were positively influenced by both personal and social motivations and physicians’ professional titles moderated this effect, with physicians with high titles emphasizing reputation and physicians with low titles emphasizing monetary rewards. Zhang et al [3] found that, when physicians reach an advanced level of expertise and knowledge, their material motivation declines and their professional motivation increases. Some physicians place great emphasis on personal branding, and their online services are designed to support their brand positioning and identity. Zhang et al [82] indicated that the OHC environment impacts brand performance, including trust and reputation, which become more significant factors in determining whether physicians participate in a consultation.

##### Economic Returns

Most physicians provide OMC services for financial reasons. OHCs need to understand how to improve financial rewards for physicians to retain good physicians. Ren and Ma [17] investigated the factors influencing physicians’ economic income in OHCs in the context of the pandemic. They found that service quality had a significant positive effect on physicians’ economic returns. In addition, they found that physician teams increase income with disease privacy and physicians who established a team were more likely to earn more money. On OHCs, physicians share articles about health and medicine as well as providing paid OMC services. According to the literature [3], physicians share free messages due to both material and professional motivation, with the role of material motivation diminishing as physicians gain more expertise. Zhang et al [78] reported that mutual aid and altruism can positively influence the willingness of health experts to share knowledge. In addition, reputation and self-efficacy can play a greater role than regular users in health experts’ willingness to share knowledge. Yang et al [81] demonstrated that physicians are motivated to share paid messages for a variety of reasons. External motivation, enjoyment motivation, and professional motivation are all important factors.

##### Social Rewards

According to the literature [16], social rewards have less influence on physician motivation than financial rewards. A combination of psychological and material rewards increases physician motivation to participate in OHCs. Material rewards are usually more useful than psychological rewards, but extreme rewards are less effective than moderate rewards. To increase physician retention, OHCs often include gamification elements such as badges, points, and leaderboards. Liu et al [83] observed that including gamification elements in medical communities can encourage continued participation and increase physician incomes, but on the other hand, gamification elements can also lead to greater income disparities among physicians.

### 2-Sided Matching

**Figure 8:**
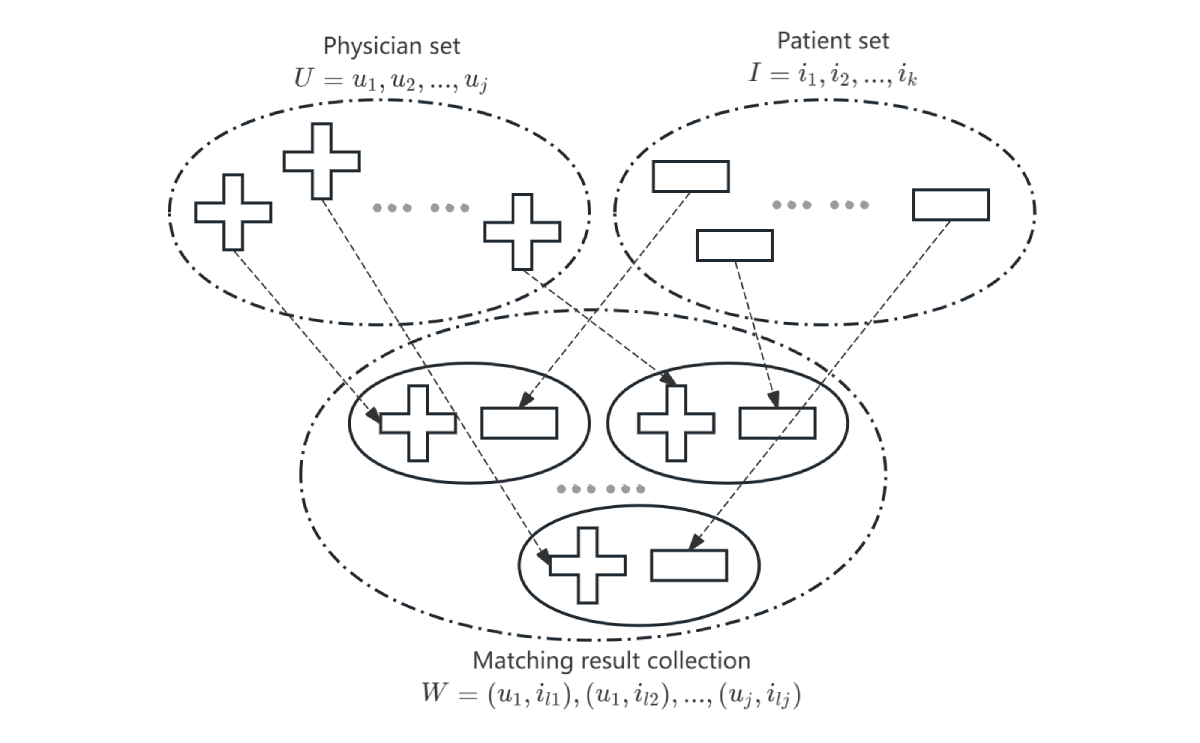
Service-oriented recommendations aim for more than just listing physicians for patients; they seek comprehensive solutions that benefit both parties. This concept is illustrated in a physician-patient matching diagram.

Unlike previous studies, this paper focused on the personalized service recommendation system for 2-sided users. It is not just about providing patients with a list of physicians but also about exploring the overall combination solutions with optimal mutual benefits for both patients and physicians, shown in Figure 8. Several important issues need to be addressed by researchers in this field, including the adoption of appropriate decision methods that effectively match the interests and preferences of both physicians and patients, improve the adoption rate of recommended solutions, and enhance the satisfaction of 2-sided users [84]. Xi and Juan [84] addressed the real problem of matching the supply and demand of health care services under an intelligent platform and proposed a decision-making method that considers both provider’s and consumer’s expectations as well as the psychological characteristics of hesitation and uncertainty. Gao et al [85] analyzed the problem of matching decisions for medical services in OHCs and constructed a matching decision model that is both satisfactory and stable. Zhong and Bai [86] analyzed the patient-physician preference matrix and constructed a 2-way matching model for specialty outpatient appointments oriented toward satisfying patients and physicians. Yang et al [87] used the 2-sided matching theory to design a patient-specialist paired appointment system in which the appointment process and the one-to-many appointment-matching algorithm were described. Chen et al [88] developed an innovative multi-attribute decision-making method for 2-sided matching considering the psychological behaviors of matching bodies as well as values of aspiration levels and evaluations.

**Figure 9:**
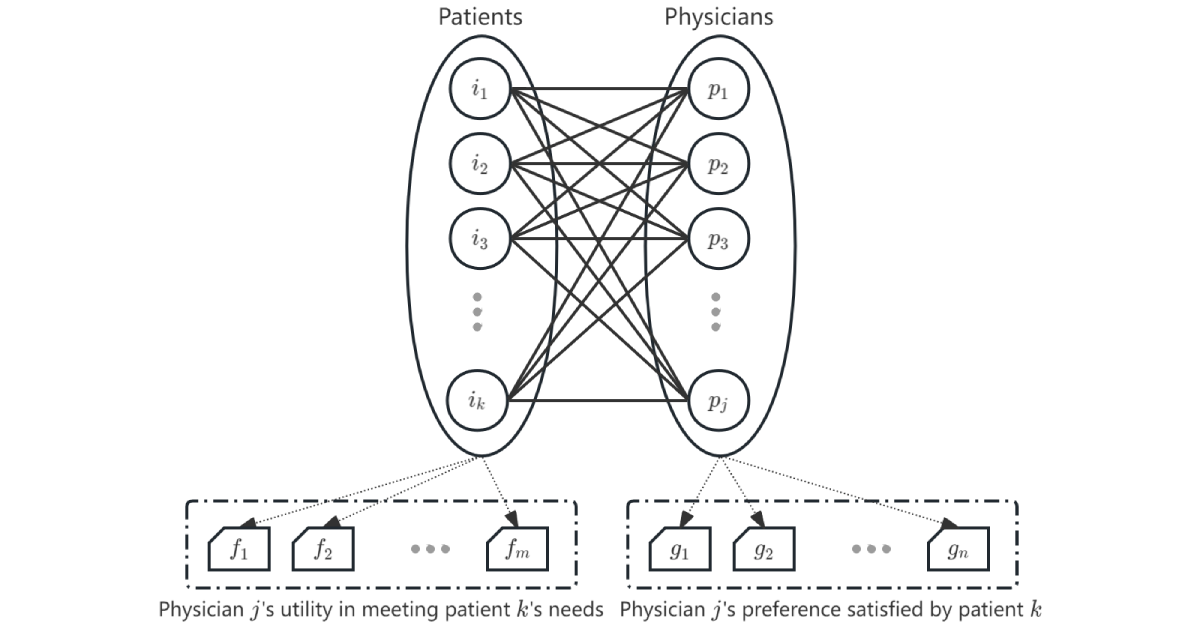
Modeling of physician-patient personalized preference order maximization. The recommendation system transforms an intuitionistic fuzzy set matrix into a satisfaction matrix and constructs a multi-objective matching model. It aims to maximize physician-patient matches while ensuring stability and satisfaction.

The future research direction of the physician-patient 2-sided matching recommendation system should take into account the decision-making environment of realistic situations. As an example, due to the complexity of medicine and the ambiguity of human thinking, most patients are unable to express clear preference sequences due to their cognitive limitations. By mining consultation text and behavioral characteristics of OHC users, the OMC recommendation system should be able to capture customized preference sequences. Even for physicians, who have higher cognitive levels, more logical behavior, and clearer motivation, there are still situations in which expectation evolution and multiple preferences cannot be ordered. Therefore, the recommendation system must accommodate their intuitive fuzzy preferences. Using intuitive fuzzy preferences, biased order relations can be expressed and preference strengths can be differentiated. Figure 9 illustrates how an intuitionistic fuzzy set matrix is transformed into a satisfaction matrix. The system should then construct a multi-objective optimized, stable 2-sided matching model based on intuitionistic fuzzy number information with the objective of maximizing physician-patient matches, stability, and satisfaction with the matching results.

### Workload Balancing

Physicians, as humans, have not only individual drivers and preferences but also variability in load tolerance. The fact that the recommended physicians represent a limited human resource has generally been overlooked in previous studies. Physicians should not overwork, and they should not be overused for an extended period [37]. Physician overload affects physician fatigue and consultative quality as well as patient waiting time, which deteriorates the comprehensive evaluation of the recommendation system [29]. Currently, very few studies have explicitly considered the workload of recommended physicians in recommender systems. To address the problem of unbalanced use among physicians, Pan et al [29] added a balanced use approach (use balancing) to a preference-learning algorithm that included a negative penalty term for physicians whose current use exceeded the mean value. To balance patient preferences and hospital staff workload, Wang et al [37] developed a utility-diversity trade-off model based on physician capacity, patient preference, and outpatient workload, which had the effect of reducing the workload for highly regarded hospitals and physicians. Yuan and Deng [4] suggested that limiting the number of times that a physician is recommended could balance the workload while exposing more people to new physicians who could also share the workload. In addition to reducing the workload of chief physicians, Yang et al [32] increased the number of recommendations to new physicians, which translates to saving time and money for patients. The system could also be used to identify the activity of each physician’s intake based on historical consultations obtained from OHCs, which, we believe, represents a difference in the upper limit of the workload of individual physicians, which is influenced by the physician’s age, specialty department, and the number of offline consultations they have received.

The load balancing of OMC service recommendations is similar to personalized reranking, which generally refers to ranking items in the recommendation result list based on the user’s preference. On the basis of the recommendation results list, load balancing attempts to determine the workload of each recommended physician, adjust the list order, or replace the candidate physicians according to their predefined individual thresholds so that the recommendations are achieved as efficiently as possible. The reranking algorithms typically use 2 categories of indicators. First, they integrate the reranking indicators directly into the recommendation algorithm to train a multi-objective model. Second, heuristics are used to optimize the reranking indicators using a 2-stage approach of filtering and reranking followed by optimization of the load balancing. Among the integrated algorithms, Adomavicius [56] presented heuristic neighborhood techniques and matrix decomposition techniques to generate a more diverse set of recommendations with a lower workload for each physician. Pedronette and Torres [89] proposed a method for reordering image content retrieval systems that combined recommendations with clustering and encoding context through ranking lists. Among the 2-stage algorithms, Yu et al [90] investigated the relationship between recommendation accuracy and diversity and proposed an adaptive trust-aware recommendation model to improve cold-start and long-tail items. In the literature [33], a dynamic exponential inventory-balancing algorithm for recommendations is presented based on the condition that physician resources are limited in a dynamic environment and based on real-time remaining resources. Wang et al [37] developed 2 heuristic algorithms for balancing patient preferences and hospital staff workload as well as updating physician rankings without changing physician capabilities so that patients can access more skilled physicians in more hospitals. In summary, the algorithms differ depending on the application scenario. On the basis of mining historical data, we can determine physicians’ work tolerance levels; to optimize recommendation results, we can personalize constraints on physicians’ upper limit of workload and dynamically optimize between patients’ needs and physician energy so that the results are maximized while maintaining the quality of recommendations and reducing the workload of physicians. Using these ideas can reduce the waiting time for patients and ease the strain on physician resources.

### Privacy Protection Issues

National legislation to protect user privacy in the health care sector is among the most stringent [30,80]. OMC service recommendations can only use anonymized, scrambled, encrypted, and other technically processed historical data. Consequently, it is difficult to obtain an individual identifier for each patient in the data set, which limits the algorithmic mining of patient features. Furthermore, national regulations regarding the prevention of leakage and misuse of personal information are becoming increasingly strict, and all personalized recommendation systems must and can only conduct legitimate research following user privacy protection [63]. Technically, CF models are not suitable for OMC recommendation scenarios regardless of whether they are user-based CF or term-based CF. A user is unlikely to seek help on the web unless they are ill or experiencing certain symptoms. Therefore, the specialty of physicians that patients seek is not determined by their explicit or implicit interests but rather by their medical needs at that time. The concept of “inferring future needs from patients’ historical data” is not logical in the context of the OMC service scenario. Unfortunately, some existing studies continue to attempt to mine peripheral information and even private information from patients, which is both illegal and ineffective. Simply reusing CF from e-commerce recommendations and recommending physicians based on historical patient data regardless of medical privacy will ruin personalized e-service recommendations. Xu et al [30] proposed an effective and privacy-preserving medical service recommendation scheme that identifies patients’ demands with physicians’ information along with their reputation score, and it is considered the first study to develop a physician recommendation scheme that ensures computational efficiency. Similarly, to ensure patient privacy, Narducci et al [25] constructed a semantic recommendation system that does not link the health data entered by patients to their true identities. As user information is protected by regulations, patient consultations contain only isolated texts and graphics related to disease descriptions. Additional information is lacking, potential preferences are unclear, and invisible needs are not addressed comprehensively. As a means of achieving intelligent recommendations under privacy protection, the system must “dance with shackles on.” To guide personalized preference mining, engineering psychology theories would be better applied, followed by natural semantic processing tools, topic models to refine patient descriptions, and semantic mining to quantify qualitative indicators. Patients’ social networks and multimodal interaction sessions in OHCs would be better collected through this system, as well as identifying potential preferences, qualitative indicators, quantitative indicators, and perceptions of patients through natural language processing, multimodal data analysis, and heterogeneous dynamic network mining.

### Contributions and Limitations

#### Theoretical Contributions

This review highlights a significant gap in research regarding service-oriented recommendations within OHCs. While OMCs are widely used on the internet, there is a notable scarcity of corresponding research on service recommendations within these environments. Traditionally, research on OMC recommendation systems has followed the conventional e-commerce model, focusing on recommending “items” to “users” rather than customizing e-service recommendations, such as recommending “users” to “users.” This lack of focus on personalized service recommendations limits the potential for enhancing user experience within OHCs. Moreover, existing recommendation algorithms primarily focus on mining, modeling, and matching expert knowledge, neglecting the consideration of 2-sided user preferences and the workload of service providers. This oversight can result in recommendations that do not effectively cater to the needs and preferences of both service providers and consumers within OHCs.

Another crucial aspect highlighted in this review is the limited consideration given to the cognitive capabilities of service consumers. Current recommendation algorithms often fail to adequately address the issue that service consumers may lack professional cognitive capabilities. Adopting interpretable recommendation algorithms could help bridge this gap and improve the effectiveness of service recommendations within OHCs. Furthermore, this review emphasizes the importance of using consumer comments judiciously in the recommendation process. While consumer comments can provide valuable insights, they should be analyzed with caution to ensure the reliability and relevance of the recommendations generated.

In summary, research on personalized recommendations for online knowledge services within OHCs is still in its early stages, facing challenges such as the “cold start” problem and the lack of a theoretical framework or algorithm. Addressing these challenges is crucial for advancing the field and enhancing the quality of service recommendations within OHCs.

#### Practical Enlightenment

The practical implications of the review findings are 2-fold and can greatly benefit stakeholders within OHCs. First, the insights provided by this review can aid OHC stakeholders, including platform administrators and policy makers, in evaluating and optimizing the design of recommender systems. By understanding that service-oriented recommendation systems should function as 2-sided matching systems rather than just expertise retrieval systems, stakeholders can make informed decisions about system design and implementation. This understanding can lead to the promotion of policies that prioritize the consideration of 2-sided preferences, thereby enhancing user satisfaction and engagement within OHCs.

Second, the review findings can assist developers in prioritizing their work and implementing measures to address key challenges faced by OHCs. For instance, developers can focus on enhancing workload balancing for physicians by optimizing recommendation algorithms to consider both the workload of service providers and the preferences of service consumers. In addition, developers can implement measures to protect patient privacy while still providing personalized recommendations, thereby fostering trust and confidence among users.

Overall, the practical value of the review findings lies in their ability to guide stakeholders and developers in optimizing the design and functionality of recommender systems within OHCs, ultimately leading to improved user experiences and outcomes.

#### Limitations and Future Work

The primary limitation is the relatively small number of included studies, leading to less robust synthesized results. Despite a growing body of research on physician recommendations, there remains a scarcity of strictly designed OMC-oriented recommender systems. Notably, while online medical applications are widely used in China, this review excluded papers published in Chinese due to language constraints.

### Conclusions

Recent years have seen an explosion of interest in physician recommendations, largely driven by the spread of OHCs and the success of artificial intelligence in other fields. As a result of the emergence of OMCs, an online service, physician recommendations have moved into a new age. These new-generation recommendation systems are service oriented rather than commodity oriented and build on the concept of 2-sided markets. This synergizes both patients and physicians with their needs and preferences individually, inspiring e-service recommendation thinking, vision, paradigms, approaches, and practices. This study has a distinctive pioneering character, and it is expected to open up a new branch of recommendation system theory. The e-service–oriented recommendations demonstrate their transformational, transdisciplinary, and translational features in terms of thinking, paradigms, methodologies, technologies, engineering, and practices. The paradigm shifts and directions are discussed in this paper. Unlike traditional e-commerce recommendations, e-service recommendations emphasize big-picture, outside-the-box thinking as well as data-driven, model-based, 2-sided hypotheses that pursue foundational and original recommendation thinking, theories, and practices from the essence of knowledge- and labor-intensive services inherent in the knowledge economy.

## Appendix

Multimedia Appendix 1 PRISMA (Preferred Reporting Items for Systematic Reviews and Meta-Analyses) 2020 checklist.

## Abbreviations

CF: collaborative filtering
GRADE: Grading of Recommendations, Assessment, Development, and Evaluations
KG: knowledge graph
LDA: latent Dirichlet allocation
OHC: online health community
OMC: online medical consultation
PRISMA: Preferred Reporting Items for Systematic Reviews and Meta-Analyses
Q&A: question and answer

## Data Availability

The data sets generated during and analyzed during this study are available from the corresponding author on reasonable request.
